# Hernioplasty and testicular perfusion

**DOI:** 10.1186/2193-1801-3-107

**Published:** 2014-02-21

**Authors:** Osman Nuri Dilek

**Affiliations:** Department of Surgery, Izmir Katip Celebi University, School of Medicine, 35620 Cigli, Izmir, Turkey

**Keywords:** Hernioplasty, Testis, Spermatic cord, Atrophy, Perfusion, Mesh

## Abstract

Open and laparoscopic tension-free techniques of hernia repair using synthetic meshes are a well-accepted practice with an excellent patient comfort and a low recurrence rate. It is well known that, direct contact of the mesh to the vessels in the inguinal canal and perimesh fibrosis may have a negative impact on testicular flow. Whether different operative techniques or mesh materials used have an effect on the integrity of the testicle, the influence of the resulting fibrosis on testicular perfusion, and spermatic cord structures is still unclear.

Our objective is to review whether there is an association between inguinal hernia and hernia repair techniques, pitfalls of various manipulations and also specific applications on spermatic cord structures and testicular perfusion in view of the literature.

Most of the clinical and experimental studies result support the idea that inguinal mesh application for hernioplasty is still a safe procedure in patients. Testicular blood flow may be influenced during open and laparoscopic inguinal hernia surgery. But, so far, there is no evidence for a significant impairment of cord structures after open or laparoscopic hernia repair using tension free techniques. It is clear that fine surgical dissection and reconstruction, doing respect for anatomy and using proper prosthetic material could be obtain the best results. Whether changes in flow parameters remain in the late postoperative period, and whether they have an impact on complications should be evaluated in further clinical and experimental studies.

## Introduction

### Surgery and anatomic structures

About 10% of people develop some type of hernia during their lifetime. In the USA, more than 750,000 hernia operations are performed each year. Hernias are seven times more common in males than in females (Fitzgibbons et al. 2005). Abramson reported that the overall current risk for a male to have an inguinal hernia was 18% and the lifetime risk was 24% (1978).

Anatomy of the spermatic cord has been well studied because of its important role in testicular physiology and surgery. The spermatic cord is composed of the vas deferens, testicular vessels including testicular artery and veins, autonomous nerves, spermatic muscle and fascia (Abramson et al. 1978; Brooks 2007). Each of these structures could be various effects on testicular perfusion. The testicular arteries arise from the abdominal aorta just below the renal artery and travel in the intermediate stratum of the retroperitoneum to reach the internal inguinal ring and become a component of spermatic cord (Brooks 2007; Dilek 2010). Beck et al. reported an anatomic study of over 100 spermatic cords identified a single internal spermatic artery in 50% of cases, with two arteries in 30% of spermatic cords and three arteries for 20% (Brooks 2007). At the internal ring, the vessel are joined by the genital branch of the genitofemoral nerve, the ilioinguinal nerve, the cremasteric artery, the vas deferens and its artery. Human testicular parenchyma is provided with approximately 9 mL of blood per 100 g of tissue per minute. Silber showed that an interruption of the testicular blood supply may result in testicular atrophy (Silber 2000).

The testicular veins (spermatic veins) form several highly anastomotic channels that surround the testicular artery as the pampiniform plexus. This arragement allows countercurrent heat exchange, which cools the blood in the testicular artery (Brooks 2007). The vascular arrangement in the pampiniform plexus, with the counterflowing artery and veins separated only by the thickness of their vascular wall in some areas, facilitates the exchange of heat and small molecules. The countercurrent exchange of heat in the spermatic cord provides blood to the testis that is a specialized structure that functions optimally 2°C to 4°C lower than the rectal temperatures in normal men (Dilek 2010; Silber 2000; Junge et al. 2011). A loss of the temperature differential may lead to testicular dysfunction (Abramson et al. 1978; Mieusset et al. 1987). Junge et al. showed that thermographic measurements at the testicle showed a significantly increased temperature in all groups including open (Lichtenstein) and laparoscopic hernia repair techniques compared to preoperative measurements. They also noted that both the technique and the mesh material have an impact on integrity of spermatic cord and testicular function (Junge et al. 2011).

The cremasteric muscle is one of the part of the spermatic cord. When this muscle contracts, the cord is shortened and the testicle is moved closer up toward the body, which provides slightly more warmth to maintain optimal testicular temperature. When cooling is required, the cremasteric muscle relaxes and the testicle is lowered away from the warm body and is able to cool. This phenomenon is known as the cremasteric reflex. The dartos muscle is a sympathetically innervated dermal muscle layer within the scrotum, distinct from the somatically innervated cremasteric muscle. Abnormalities of dartos and cremasteric muscle innervation may impact testis thermoregulation and spermatogenesis (Silber 2000; Kurz & Goldstein 1986; Mihalache et al. 1996).

Autonomous nerves reach the testis accompanying the testicular artery and pampiniform plexus. The vast majority of testicular nerves are sympathetic axons with vasomotor function and innervate the small vessels supplaying cluster of Leydig cells and regulate testicular LH receptors and blood flow (Schlegel et al. 2007).

Anatomically, there is a closed relation between the spermatic cord structures and inguinal hernias. Inguinal hernias can carry the risk of ischemia of the testis by an intermittent mechanical compression (pressure) on the testicular vessels (Fitzgibbons 2007; Turgut et al. 2007). In some report, color doppler ultrasonography showed that preoperatively there was a significant elevation in the sonographic resistive index (RI) in the affected (hernia) side compared with the normal side (Beddy et al. 2006). On the other hand, Munoz Sanchez concluded that the uncomplicated inguinal hernias do not cause any significant alterations in the arterial circulation of the testicle (Abramson et al. 1978; Muñoz Sánchez et al. 2005).

### Hernioplasty and testicular damage

Testicular damage (atrophy and/or dysfunction) is one of the most dreaded sequela of inguinal hernioplasty. However, literature findings show that testicular atrophy occurred in 0% to 2% of patients after hernioplasty (Fitzgibbons 2007; Turgut et al. 2007; Lee et al. 2000). Yavetz et al. reported that among 8500 patients attending the fertility clinic due to infertility, 565 men (6.65%) reported an incidence of inguinal hernioplasty with or without subsequent atrophy of the testis (Yavetz et al. 1991).

The laparoscopic totally extraperitoneal preperitoneal hernia repair (TEP) technique, which is based on the concept of tension-free high ligation of the sac, has become widely popular in surgical practice (Stoppa & Waarlaumont 1989; Nyhus et al. 1960; Kingsley et al. 1998; McKernan & Laws 1993). The Lichtenstein hernia repair (LHR) is one of the most comfortable effective methods of inguinal hernia repair, and it has similarities with TEP because of the prosthetic mesh (Collaboration EH 2000). Despite the frequency of open and laparoscopic herniorraphy the effect of the hernia and subsequent repair on testicular perfusion and function is unknown. It is unclear at present what the best method is among mesh implantation, reconstructing the deep inguinal ring in hernia surgery (Dilek 2010).

The preoperative and postoperative use of color duplex ultrasonography (CDUS) to evaluate the spermatic cord structure and scrotal structure has been well documented in testicular pathologies and hernias (Fitzgibbons 2007; Ponka 1980; Oyen 2002). CDUS is extremely helpful in all cases to investigate extratesticular vascularization and testicular perfusion, with parameters optimized to display low flow velocities including peak systolic velocity (PSV), end diastolic velocity (EDV). Lefort et al. showed that examination of the scrotum with CDUS should include measurement of intratesticular resistive index (RI) and elevated RI can be suggestive of ischemia (Lefort et al. 2001).

In our prospective randomized series of 82 patients (Akbulut et al. 2003; Dilek et al. 2005), blood flow parameters of the spermatic artery were evaluated and noted as peak systolic velocity (PSV), end diastolic velocity (EDV), and calculated resistive index (RI). We could not find any significant changes in blood flow parameters (PSV, EDV, and RI) when comparing both groups pre- and postoperatively. Also, there were no significant changes in the blood flow parameters when the TEP and LHR groups were compared to each other (Akbulut et al. 2003; Dilek et al. 2005). We did not find any significant differences between the techniques on blood flow in the testes. Our previous report included the same population, and neither TEP nor LHR affected testicular function, but TEP decreased testicular volume but in normal limits (Dilek 2010; Akbulut et al. 2003; Dilek et al. 2005).

## Discussion

The aim of all inguinal hernia repair techniques is to close the internal ring with a suture or a biomaterial such as polypropylene mesh. The matter has been raised whether or not the spermatic cord structures are compromised with these techniques. The spermatic cord structures may be exposed to invasive surgical intervention during inguinal hernia reconstruction. Surgical dissection, division, or mechanical trauma to the spermatic artery and veins account for serious trophic changes in the testis. Lee et al. explained that surgical manipulation of the spermatic cord imparts a small, but statistically significant morphological change in testicular size without a deleterious effect on testicular development, fertility, or fecundity (Lee et al. 2000).

There are many factor lead to decrease and/or interruption of the testicular perfusion (Segenreich et al. 1997 Sigman et al. 1997 and Wantz 1986). In some reports, inguinal hernia may impair testicular blood flow, which may be attributable to an intermittent mechanical compression effect on the funiculus spermaticus in the inguinal canal (Dilek 2010; Fitzgibbons 2007; Turgut et al. 2007; Beddy et al. 2006; Akbulut et al. 2003). Testicular artery and vein injuries, thrombosis of spermatic vein plexus, testicular torsion are the major factors influencing the testicular perfusion. Furthermore, the implantation of a non-absorbable polypropylene mesh during hernia repair causes chronic foreign body reaction involving the surrounding tissue. In case of inguinal hernia repair using different mesh techniques, the spermatic cord structures is potentially affected by this chronic inflammatory tissue remodeling (Peiper et al. 2005). However, there are many authors reported that the testes has more vessels than expected. Testicular arterial anatomy has been well studied because of its important role in testicular physiology and testicular surgery. Anatomically, the spermatic artery divides into two branches near the testis. Jarow et al. showed that the frequent early branching of the internal spermatic artery will prevent inadvertent interruption of testicular arterial blood flow during operations performed upon the spermatic cord within the inguinal canal (Jarow et al. 1992). The testicular artery penetrates the tunica albuginea at the lower pole, proceeding as the capsular artery. Using CDUS, a transmediastinal artery is visible in the upper third of the testis in 50%. Branches from the capsular artery course through the parenchyma in the testicular septations as afferent arteries and are directed to the gonadal hilum. The testicular veins are not consistently visible using CDUS (Oyen 2002).

**Figure 1:**
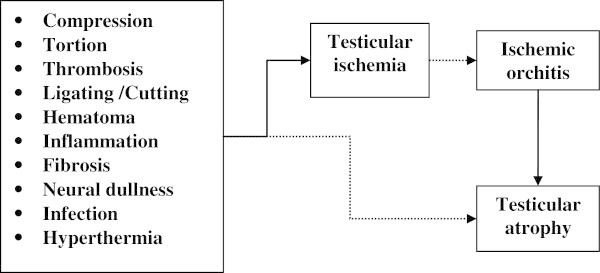
**Ischemic conditions of testis and its relation with the testicular perfusion and testicular atrophy.**

Many studies suggest an unknown or alternative (collateral) connections between vessels of the cord and other vessels that supply blood to the testis (Fitzgibbons 2007; Muñoz Sánchez et al. 2005; Moore & Hasenboehler 2007; Zomorrodi & Buhluli 2008; Zát’ura et al. 1997). Zomorrodi and Buhluli explained that they isolated and ligated the spermatic cord at the internal ring of the inguinal canal for transfixation and placing the allografted kidney in retroperitoneal position with anastomoses of the iliac vessels, and mass ligation of the spermatic cord did not lead to any ischemic problems in the follow up period (Zomorrodi & Buhluli 2008). Zát’ura et al. concluded that in the great majority of men the blood supply of the testis is ensured by collateral circulation (Zát’ura et al. 1997). It is well known that compression, thrombosis, ligating and/or cutting of the spermatic vessels may lead to ischemia, ischemic orchitis and testicular atrophy (Figure 1). Ischemic orchitis typically presents 2–3 days after inguinal hernia surgery and can progress to infarction. This ischemic injury is likely due to thrombosis of the venous plexus, rather than iatrogenic arterial injury or inappropriate closure of the inguinal canal (Dilek 2010; Moore & Hasenboehler 2007). Venous outflow obstruction secondary to thrombosis of the pampiniform plexus can also cause testicular infarction as a result of overzealous dissection of the cord or excessive use of diathermy; it may also be the result of pressure from a large hematoma in the groin (Wantz 1982).

Testicular torsion is also very important and unpleasant problem, and significantly reduces testicular vascular perfusion. Turner et al. reported that in an experimental study, experimental torsion significantly reduced testicular vascular perfusion. Five minutes after torsion repair, the mean flow values had returned to approximately 70% of the pretorsion values. Testicular torsion significantly reduced the venous plasma testosterone concentrations at both 3 and 30 days after torsion repair. They suggest that reperfusion/oxidative stress may play a role in Leydig cell dysfunction, as well as by acting directly in germ cell apoptosis (Turner et al. 2005). Testis perfusion can be maintained for a prolonged period in the presence of testicular torsion. Anatomical variability may account for differences in the duration of viability of the torsed testis (Bentley et al. 2004). It is clear that the breakage of testicular perfusion can lead to testicular damage (atrophy). There are also another reasons such as obstruction of vas deferens, inguinal hematoma, infections and immunological reactions (Fitzgibbons 2007; Oyen 2002; Shin et al. 2005).

**Figure 2:**
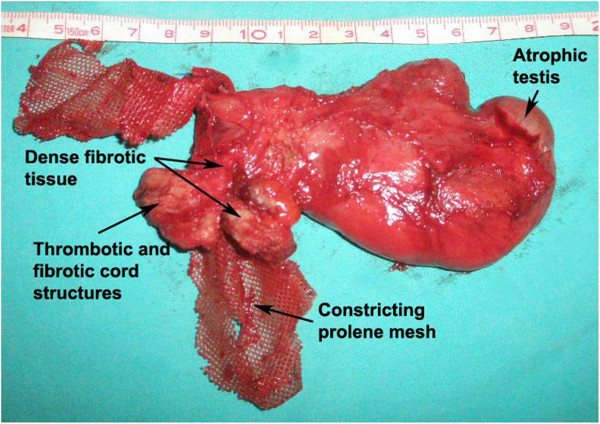
**Prostetic mesh induces a chronic foreign-body reaction involving the surrounding tissue and the spermatic cord structures and could be impair the testicular perfusion and finally, lead to testicular atrophy.**

Prosthetic mesh implantation is regarded as the standard treatment of inguinal hernias. For about 25 years the use of prosthetic materials in the repair of inguinal hernias has been routine in general surgery. An estimated 80% of inguinal hernia operations involve placement of a prosthetic mesh to form a “tension-free” hernioplasty (Dilek 2010). The prosthetic mesh induces a chronic foreign-body fibroblastic response creating scar tissue that imparts strength to the floor and leads to fewer recurrences (Shin et al. 2005). The use of prosthetic materials for inguinal hernia markedly reduces the recurrence rates, postoperative hospital stay, pain and discomfort. But, the protheses adhere frequently to the cord structures in most cases. The disadvantages are local wound complications, technical difficulties in hernia repair, restriction of mobility by the rigid shell, contraction of mesh (Figure 2), and the complications related with cord structures such as varicocele, hydrocele, ischemic orchitis, testicular atrophy, and finally infertility (Dilek 2010; Homonnai et al. 1980).

In the literature, studies comparing the effect of different techniques using by mesh prostheses are rather limited. The influence of the Lichtenstein and Shouldice operations on the cord structures in a canine model was investigated and no significant differences with regard to testicular volume and blood flow were found between the operation groups or between the pre- and postoperative results (Homonnai et al. 1980; Uzzo et al. 1999). Many clinical studies reported that the similar results in which the choice of either Lichtenstein or TEP hernia repair technique did not significantly alter the testicular function. Patients with inguinal hernia have an elevated testicular vascular resistance, which is reversed after repair. The choice of laparoscopic or open herniorraphy did not affect reversal of this surrogate of testicular function (Beddy et al. 2006; Uzzo et al. 1999). Laparoscopic inguinal hernia repair using suture closure of the internal inguinal ring does not impair testicular perfusion. The advantages of the laparoscopic approach also include that its technical ease, it is an outpatient procedure, the cord structures remain untouched, the type of hernia is obvious, clear visualization of the anatomy (Dilek 2010; Schier 2006). However, some clinical and experimental studies also revealed that a dense fibroblastic response encompassing the polypropylene mesh with either trapped or obliterated the testicular vessels and vas deference (Peiper et al. 2005; Shin et al. 2005). Peiper et al. reported that the implantation of a non-absorbable polypropylene mesh in the inguinal region during hernia repair causes chronic foreign body reaction involving the surrounding tissue and the spermatic cord structures in pigs. They observed that venous thrombosis of the spermatic veins occurred in five of 15 cases. The mesh repair may also lead to a decrease of arterial perfusion, testicular temperature, and the rate of seminiferus tubules with regular spermatogenesis. Therefore, they recommend strict indications for implantation of a prosthetic mesh during inguinal hernia repair (Peiper et al. 2006).

On the other hand, prosthetic materials can contract by 20-75% of its original size within one year after implantation in the inguinal region. Taylor et al. set out to determine whether this contraction has any effect on testicular or femoral vessel blood flow following open or laparoscopic hernia repair. They found that mesh contraction following inguinal hernioplasty does not adversely affect the testis or femoral vessels and can be used safely for both anterior and preperitoneal approaches (Taylor et al. 2001).

Testicular perfusion following hernioplasty can be easily monitored and evaluated with Duplex ultrasonography, the flow in the spermatic artery and testicular artery and its branches is of low resistance, with a relatively broad systolic part and holodiastolic flow. CDUS enables a definitive diagnosis of ischemia and decreased testicular circulation. A pitfall to remember in the diagnosis is that hypervascularity can occur (Brisinda et al. 2008). Testicular and epididymal swelling along with a slightly decreased echogenicity have been reported to develop in the first hours, although in most cases the hypoechogenicity occurs later, so that examining the testis 3 months after the operation seems to be more rational as was performed in our study (Taylor et al. 2001). Brisinda et al. also found that there were no statistically significant differences between preoperative and postoperative measurements which included testicular blood flow parameters and testicular volume (Brisinda et al. 2008).

As a result; there are many clinical and experimental study results in which has advers effect or not on testicular perfusion (Lima Neto et al. 2007; Koksal et al. 2010). We thougt that it is difficult to impair the testicular perfusion after hernia repair, because of it has rich arterial supplay and collateral capacity. Arterial input and venous drainage of the testis are assured by many anastomoses, which protect it from ischemic injury (Lefort et al. 2001; Reid & Devlin 1994). Careful dissection and preservation of the vessels are important to protect these anastomoses during hernia repair. The idea that deep ring repair that is too tight may cause testicular ischemia is erroneous.

## Conclusions

### Surgical pitfalls; prevention and management

There are many application to protect the testicular perfusion. Surgeons should be trained that all inguinal hernias should be repaired at diagnosis, even if asymptomatic. Giving information about the hernias and its complications should be more usefull for the public. This treatment algoritm will decrease and prevent the complications such as incarceration, strangulation and that hernioplasty becomes more difficult the longer repair is delayed (Fitzgibbons et al. 2005; Fitzgibbons 2007).

**Suggestions to protect the testicular perfusion**

✓ Training, education and information.

- Surgeon
- Patient and Family

✓ Dissection

- Respect to anatomy
- Minimized trauma
- Limited dissection
- Never dissect beyond the pubic tubercle
- Leave distal hernia sacs
- Save the cremasteric muscle fibers
- Reconstruct the cremasteric fascia

✓ Recurrent hernias;

- Avoid dissection
- Prefer Properitoneal (Posterior) approach

✓ Mesh fixation

- Fibrin glue
- Selfadhesive mesh

✓ Avoid mesh aplication

- Young reproductive age
- Patients with solitary testis

✓ Testis

- Do not dislocate the testis from the scrotum into the wound
- Avoid concomitant scrotal surgery.

Trauma to structures of the spermatic cord should be minimized and the incidence of testicular atrophy could be reduced by limiting dissection trauma to the spermatic cord; never dissecting beyond the pubic tubercle; leaving distal indirect hernia sacs attached to the cord; as in recurrent hernias, avoiding dissection of the spermatic cord altogether by employing a posterior properitoneal approach (Wantz 1982). Preserving the cremasteric muscle fibers and reconstructing the fascia can protect the structures of the spermatic cord from the inflammatory reaction (Mihalache et al. 1996; Peiper et al. 2006).

Overzealous dissection of a distal hernia sac, dislocation of the testis from the scrotum into the wound and concomitant scrotal surgery should be avoided (Reid & Devlin 1994).

The polypropylene mesh hernioplasty needs to be carefully advised of potential obstruction and compromise to future fertility in men, especially of young reproductive age or with a solitary testicle (Shin et al. 2005).

Properitoneal repairs should be considered for repairs of recurrent hernias not only to reduce further recurrences but also to avoid testicular complications. Using an intraperitoneal onlay composit mesh and the safety of its fixation with fibrin glue could be an alternative in the treatment of recurrent and complicated inguinal hernias (Olmi et al. 2007).

General opinion is that neither open nor laparoscopic hernioplasty techniques do not affect the testicular circulation. It is clear that fine surgical dissection and reconstruction, doing respect for anatomy and using proper prosthetic material could be obtain the best results. To be consistent, future animal and clinical studies have to be performed in large groups and focus on the use of mesh, which may increase the intensity of the mesh reaction to the cord structures.
